# The Effects of Small-Volume Liposuction Surgery of Subcutaneous Adipose Tissue in the Gluteal-Femoral Region on Selected Biochemical Parameters

**DOI:** 10.3390/ijerph16183298

**Published:** 2019-09-08

**Authors:** Anna Lubkowska, Monika Chudecka

**Affiliations:** 1 Department of Functional Diagnostics and Physical Medicine, Faculty of Health Sciences Pomeranian Medical University in Szczecin, Żołnierska 54 Str., 71-210 Szczecin, Poland; 2 Department of Functional Anatomy and Biometry, Faculty of Physical Education and Health Promotion, University of Szczecin, al. Piastów 40b/6, 71-065 Szczecin, Poland

**Keywords:** subcutaneous adipose tissue, liposuction surgery, biochemical parameters

## Abstract

Liposuction is becoming an increasingly common procedure of aesthetic surgery, that patients choose to shape the body. Apart from the risks associated with the surgery, one should also consider whether the reduction of adipose tissue can significantly affect the metabolism of lipids and carbohydrates and, indirectly, that of bone tissue. The aim of the presented study was to assess the effects of small-volume liposuction surgery in the gluteal–femoral region on the selected markers of carbohydrate, lipid, and bone metabolism. The study included 27 women (40.75 ± 13.67 years of age, BMI = 25.9 ± 4.13 kg/m^2^) subjected to the removal of 3.35 ± 0.994 L of adipose tissue to shape the body. Following the procedure, significant changes in the body composition and body adiposity indicators were observed in these women. A slight decrease in adiponectin, leptin, resistin and insulin levels and HOMA-IR value was found three months after the procedure. No changes in the lipid profile of the subjects were found. It can be concluded that the removal of a small volume of adipose tissue from the gluteal-femoral region has a slight but positive effect on carbohydrate and lipid metabolism, providing a decreased risk of developing insulin resistance.

## 1. Introduction

Liposuction can be classified as a cosmetic method to fight obesity. It is a procedure consisting in breaking down and removing specific and unnecessary resources of subcutaneous adipose tissue in healthy individuals in various areas of the body in order to correct the contour and shape of selected body parts, and to create a more balanced physique. It is one of the most commonly performed surgical procedures in aesthetic plastic surgery [1,2,3]. The development of adipose tissue during puberty is greater in women than in men. This can be explained by the effects of oestrogens because 17-beta-oestradiol stimulates the replication of adipocytes. Research shows that between 40% and 70% of adolescent girls are dissatisfied with two or more parts of their bodies, which most often include hips, buttocks, abdomen and thighs, the parts of the body that are most exposed to fat deposition [4]. Liposuction has become popular and commonly performed with few side effects, despite possible complications, the risk of which increases with increased lipoaspirate volumes (e.g., fat embolism syndrome). Each liposuction procedure leads to the destruction of parts of the blood vessels that supply the skin and innervation of the skin. Adipose tissue in the human body consists of the connective tissue stroma composed of cells and extracellular substance, mature adipocytes, blood vessels, nervous tissue, immune cell fibroblasts, and preadipocytes–precursor cells [5].

Body fat dysregulation, often characterised by changes in the distribution of adipose tissue, is associated with a wide range of conditions, including obesity, lipodystrophy, anorexia nervosa, bulimia and, consequently, endocrine and metabolic disorders.

It has been suggested that weight loss is accompanied by changes in the inflammatory state and adipokine levels, moreover the decrease in adipose tissue by liposuction could affect adipokine secretion and, hence, the metabolic profile; however, it could depend on what type of fat has been removed. Abdominal liposuction provides a unique opportunity to evaluate the importance of subcutaneous abdominal fat in the risk of coronary heart disease in persons with abdominal obesity. Research on the effects of liposuction procedure on the lipid profile, insulin resistance, pro-/anti-inflammatory markers and adipocytokine concentration are often directed towards the search for possible metabolic benefits resulting from the removal of a certain volume of fat tissue. The results, however, are still inconclusive, and, in particular, there are no results for the liposuction of the gluteal–femoral region. Some authors have claimed that liposuction does not have prominent metabolic effects in terms of insulin sensitivity and pro-inflammatory adipocytokines [6,7,8,9]. while others have reported transient benefits in a reduction of the risk of coronary heart disease [10,11]. The interpretation of data from such studies is confounded by the differentiation of the groups in terms of initial body mass and composition, liposuction area, amount of aspirated body fat, level of physical activity, weight changes that occurred among the subjects after liposuction was performed, by variations in the volume of adipose tissue removed and the site of its removal, by differences in the methods used to assess insulin sensitivity, and by differences in the subjects’ baseline weight and insulin sensitivity. However, there have been no studies performed on the association between liposuction and serum adipocytokine concentration and bone turnover and bone remodelling markers in humans which, in the context of an increase in the popularity of liposuction procedures among women, may be of great importance for the development of the risk factor for osteoporosis, especially in the perimenopausal period.

Thus, the purpose of this study was to explore the effects of a small-volume liposuction in the gluteal–femoral region on selected lipid and carbohydrate metabolism parameters in healthy women, including lipid indicators, biological agents released from adipose tissue, pro and anti-inflammatory cytokines, and osteoprotegerin, one of the most important systems in bone biology: RANKL/OPG/RANK (Receptor Activator for Nuclear Factor κ B Ligand/ Osteoprotegerin/ Receptor for RANK-Ligand) When undertaking the described studies, the question was whether the surgical removal of a specific amount of adipose tissue from selected areas of the body may be a factor significantly modifying the values of metabolism indicators in question in women who undergo this form of plastic surgery to model the body and what is the direction of potential changes.

## 2. Material and Methods

The examinations were limited to adult subjects. Twenty-seven physically inactive women (40.75 ± 13.67 years of age, height of 171 ± 7.8 cm, BMI = 25.95 ± 4.13 kg/m^2^) volunteered and were positively qualified by a physician for liposuction procedure of the gluteal-femoral region at the Aesthetic Med Andrzej Dmytrzak Plastic Surgery Clinic in Szczecin, Poland. Each of the participants) gave her written informed consent before participating in the study. The study was approved by the local Ethics Committee (Pomeranian Medical University Ethics Committee, Ref. KB 0012–15/12). This study was performed in compliance with the Declaration of Helsinki as well. All the subjects were normotensive (brachial artery blood pressure < 140/90 mmHg). The exclusion criteria were: BMI over 30 Kg/m^2^; smoking; metabolic disorders such as glucose intolerance, diabetes, hypertension, thyroid dysfunction, and dyslipidaemia; unstable body weight in the last six months prior to the commencement of the study; current use of medications including antidepressants, appetite suppressants, thyroid hormone medication, orlistat, topiramate, diuretics, anti-inflammatory, or antibiotics; and previous liposuction surgery. Anthropometric parameters and blood pressure were measured prior to (PRE) and 12 weeks after intervention, during the control visit (POST), by using standard methods. The 12-week delay was intended to eliminate the confounding effects of post-surgical inflammation on our study end-points. Body weight was measured to the nearest 0.1 kg while body height to the nearest 0.5 cm. Waist circumference (WC) was measured at the minimum circumference between the iliac crest and the rib cage at minimal respiration. Hip circumference was measured at the maximum protuberance of the buttocks. Waist-to-hip ratio (WHR) was calculated. BMI was defined as weight in kilograms divided by height in meters squared (kg/m^2^). Blood pressure was measured after at least 10-min rest in the sitting position. An electrical bioimpedance method (using a Jawon Medical X-Scan Plus II body composition analyser ( Jawon Medical Co., Ltd, Seoul, Korea) was used to estimate body composition parameters: body fat mass (BFM), subcutaneous fat mass (SFM), visceral fat mass (VFM). The subjects were instructed to resume their normal lifestyle after the recovery period.

### 2.1. Blood Sampling and Biochemical Analysis.

During the experimental period, venous blood samples were collected twice to perform haematological and biochemical analyses: Before the liposuction surgery (PRE, a day before the liposuction procedure), and three month after intervention (POST). Venous blood samples were obtained in the morning after an 8–10 h overnight fasting, between 8:00 and 9:30 a.m., from the antecubital forearm vein, after a 10-min rest in the sitting position, using the Vacutainer system tubes (Sarstedt, Germany) with appropriate anticoagulant, for determination of blood count (1.2 mL; anticoagulated with 1 g/L EDTA): Erythrocyte number (RBC), haemoglobin concentration (Hb), haematocrit value (Ht), leukocyte number (WBC), and thrombocyte number (PLT), and one dry tube (7 mL) in order to obtain blood serum. Haematological parameters were measured using an automated haematology analyser (HORIBA ABX Micros 60). After blood sample centrifugation (300 rpm, 1500 *g*, 10 min, 4 °C), serum was divided into aliquots and immediately deep-frozen at −70 °C until analysis, but not longer than one month. In the blood serum was determined—total protein, albumin, glucose, uric acid levels; and lipid profile indicators—total cholesterol (T-Ch), high-density lipoprotein cholesterol (HDL) and triglyceride concentrations (TG), with the enzymatic colorimetric method using appropriate commercial kits (BioMaxima, Poland). The low density cholesterol fraction (LDL) was calculated using the Friedewald formula [12], which can be used with TG values not exceeding 350 mg/dL:

LDL [mg/dL] = total cholesterol (TCh) − HDL cholesterol − (TG/5)(1)

Additionally, the serum levels of adiponectin, leptin, visfatin, resistin, ApoA, ApoB, ApoE, CRP, insulin, IL-1β, IL-2, IL-6, IL-10, TNF-α, TNF-β, and OPG were measured by immune–enzymatic assays using commercially available ELISA kits. The serum levels of adiponectin were measured by immune–enzymatic assays using commercially available ELISA kits (Mediagnost, Reutlingen, Germany). The adiponectin assay sensitivity was <0.6ng/mL, intra- and inter-assay CVs were 6.7% and 4.7%, respectively. The serum levels of leptin were measured by immune–enzymatic assays using commercially available ELISA kits (DRG, Frauenbergstr, Marburg). The leptin assay sensitivity was 1.0 ng/mL, intra- and inter-assay CVs were 5.95–6.91% and 8.66–11.55%, respectively. The serum levels of visfatin was measured by immune–enzymatic assays using commercially available ELISA kits (Phoenix Pharmaceuticals, Inc, CA, USA). The visfatin assay sensitivity was 1.8 ng/mL, intra- and inter-assay CVs were <10% and <15%, respectively. The serum levels of resistin was measured by immune–enzymatic assays using commercially available ELISA kits (Mediagnost, Reutlingen, Germany). The resistin assay sensitivity was 0.012 ng/mL, intra- and inter-assay CVs were 6.8% and 5%, respectively. The serum levels of ApoA-I were measured by immune–enzymatic assays using commercially available ELISA kits (AssayPro, St. Charles, IL, USA). The ApoA-I assay sensitivity was 1µg/mL, intra- and inter-assay CVs were 4.9% and 7.2%, respectively. The serum levels of ApoB was measured by immune–enzymatic assays using commercially available ELISA kits (AssayPro, St. Charles, IL, USA). The ApoB assay sensitivity was 0.0078 µg/mL, intra- and inter-assay CVs were 5.1% and 7.4%, respectively. The serum levels of ApoE was measured by immune–enzymatic assays using commercially available ELISA kits (Qayee-bio, Shanghai, China). The ApoE assay sensitivity was 0.067 ng/mL, intra- and inter-assay CVs were <10% and <15%, respectively. The serum levels of CRP were measured by immune–enzymatic assays using commercially available ELISA kits (Qayee-bio, Shanghai, China). The CRP assay sensitivity was 0.78 ng/mL, intra- and inter-assay CVs were <10% and <15%, respectively. The serum levels of insulin were measured by immune–enzymatic assays using commercially available ELISA kits (DRG, Frauenbergstr, Marburg, Germany). The insulin assay sensitivity was 1.76 µlU/mL, intra- and inter-assay CVs were 1.8–2.6% and 2.9–6.0%, respectively. The serum levels of IL-1β were measured by immune–enzymatic assays using commercially available ELISA kits (DRG, Frauenbergstr, Marburg, Germany). The IL-1β assay sensitivity was 0.35 pg/mL, intra- and inter-assay CVs were 1.4–2.3% and 2.5–4.9%, respectively. The serum levels of IL-2 were measured by immune–enzymatic assays using commercially available ELISA kits (DRG, Frauenbergstr, Marburg, Germany). The IL-2 assay sensitivity was 9.1 pg/mL, intra- and inter-assay CVs were 7.0% and 5.0%, respectively. The serum levels of IL-6 were measured by immune–enzymatic assays using commercially available ELISA kits (Qayee-bio, Shanghai, China). The IL-6 assay sensitivity was 3.12 pg/mL, intra- and inter-assay CVs were <10% and <15%, respectively. The serum levels of IL-10 were measured by immune–enzymatic assays using commercially available ELISA kits (DRG, Frauenbergstr, Marburg, Germany). The IL-10 assay sensitivity was 1.6 pg/mL, intra- and inter-assay CVs were 2.8–3.7% and 2.7–2.8%, respectively. The serum levels of TNF-α were measured by immune–enzymatic assays using commercially available ELISA kits (Qayee-bio, Shanghai, China). The TNF-α assay sensitivity was 15.6 ng/mL, intra- and inter-assay CVs were <10% and <15%, respectively. The serum levels of TNF-β were measured by immune–enzymatic assays using commercially available ELISA kits (Sunredbio, Shanghai, China). The TNF-β assay sensitivity was 0.758 ng/L, intra- and inter-assay CVs were <10% and <12%, respectively. The serum levels of OPG were measured by immune–enzymatic assays using commercially available ELISA kits (Sunredbio, Shanghai, China). The OPG assay sensitivity was 2.044 ng/L, intra- and inter-assay CVs were <10% and <12%, respectively. The insulin sensitivity was determined using the Homeostasis Model Assessment Index of insulin resistance (HOMA-IR) according to the following formula:

HOMA-IR = fasting insulin (mU/L) × fasting glucose (mmol/L)/22.5(2)

### 2.2. Statistical Analyses

The obtained results were statistically analysed. Distributions were examined using the Shapiro–Wilk test which indicated that some variables deviated from a normal distribution (they were lognormal). Each studied parameter was characterised by sample size, arithmetic mean / median, and standard deviation. The data were tested by one-way ANOVA. Since in some cases the distribution was not normal a non-parametric Wilcoxon post hoc test for a dependent variable were performed when a significant *F*-value was found. The accepted level of significance was defined as *p* < 0.05. Development of the statistical results was performed using STATISTICA PL v. 11 software (Statsoft, Krakow, Poland).

## 3. Results

The patients reporting to the plastic surgery clinic were healthy and after the positive qualification underwent a small-volume liposuction surgery of the gluteal–femoral region (mean of 3.35 ± 0.994 L harvested fat). No serious complications occurred in any subject, and all were able to return to their usual lifestyle within 14 days after liposuction.

Table 1 presents the general characteristics and mean values of haematological parameters of the examined women being evaluated before the surgery. Anthropometric and demographic characteristics of the patients before and three months postoperatively are depicted in Table 2. At baseline, all women displayed normal body weight or slight overweight; mean waist circumferences were 84 cm and WHR = 0.86. After lipoaspiration, a statistically significant decrease in the value of the indicators of all analysed morphological traits was observed.

Twelve weeks after surgery, body fat mass decreased on average by 4 kg from baseline (*p* = 0.0014), which led directly to a reduction of BMI from 25.7 to 24.5 (*p* = 0.0076). Simultaneously, liposuction caused a decrease in waist and hip circumference, which resulted in a decrease in WHR from 0.86 to 0.85 (*p* = 0.0076). Following the procedure, the content of subcutaneous fat was reduced from 18.8 to 17.3 kg (*p* = 0.0014) and that of visceral fat from 2.3 to 2.2 kg (*p* = 0.0015).

The effect of liposuction on the parameters of carbohydrate and lipoprotein metabolism, adiponectin level, as well as selected biochemical markers is shown in Table 3. As shown, liposuction did not significantly alter the concentration of total protein, albumin or glucose serum concentration. Biochemical parameters, the values of which were elevated at week 12 after liposuction, were: Uric acid (*p* = 0.0013), pro-inflammatory cytokines Il-2 (*p* = 0.0277) and IL-6 (*p* = 0.0392). As a result of liposuction, no changes in the components of lipid metabolism (HDL, LDL, TG, ApoA, ApoB and ApoE) were observed except for a slight reduction in total cholesterol (*p* = 0.0277). In addition, there was a decrease in adiponectin (*p* = 0.03741), leptin (*p* = 0.0107) resistin (*p* = 0.033) and insulin (*p* = 0.0012) levels and HOMA-IR value (*p* = 0.0159). There were no significant effects of liposuction on bone turnover markers.

## 4. Discussion

The presented studies evaluated the effects of small-volume liposuction in the gluteal–femoral region in women with normative body weight on the parameters of carbohydrate and lipid metabolism as well as the factors that may affect the metabolism of adipose and bone tissue. Excessive body weight and increased amount of body fat, especially in the abdominal area, is the main reason for the development of tissue resistance to insulin, while the distribution of adipose tissue seems to be of key importance in the development of metabolic disorders in ever younger persons [13,14,15].

As far as the function is concerned, there are clear regional differences regarding both the sensitivity to hormones and the metabolic activity of human adipose tissue. The only hormones that strongly affect the lipolysis of human adipocytes are catecholamines and insulin. Catecholamine-stimulated lipolytic reactivity is greater in visceral fat than in abdominal subcutaneous tissue, and lowest in the adipose tissue of the gluteal–femoral region [16,17]. White adipose tissue is distributed in several anatomically separate and isolated “depots”, such as the subcutaneous and intra-abdominal stores, each of which is being characterised by its own metabolic functions. Berman and his collaborators have found in their study that the gluteal femoral adipocytes are white adipocytes, as determined by their response to catecholamines, because they are predominantly positive for α2 adrenergic receptors that have anti-lipolytic effects as opposed to abdominal adipocytes, mainly containing β adrenergic receptors [18].

Adipocytes have many receptors that are responsible for their sensitivity to regulating humoral factors, and thus allow the interaction of adipose tissue with endocrine, nervous and immune systems, while regulating bone metabolism. Adipose tissue is innervated by the noradrenergic, sympathetic nervous system and is an active endocrine organ synthesising numerous biologically active peptides called adipokines as well as other factors, such as: Tumour necrosis factor (TNF-α), interleukin IL-1β, interleukin-6 (IL-6), interleukin 10 (IL-10), type 1 plasminogen activator inhibitor (PAI-1) or angiotensin II, which act in adipose tissue (autocrine and paracrine effects) as well as in distant organs and tissues (classic endocrine activity).

These compounds regulate the appetite processes that affect the energy balance of metabolic pathways as well as the inflammatory processes. Few studies have shown that adipose tissue, as a component of body weight and a direct marker of obesity, has protective effects on bone tissue. In in vitro studies, direct effects of leptin on the differentiation of bone marrow mesenchymal cells towards osteoblasts and inhibition of differentiation towards adipocytes were observed [19], while in in vivo studies it is postulated that the administration of leptin peripherally enhances the bone formation [20] and inhibits osteoclast activity and bone resorption, by inhibiting RANK synthesis, the RANK ligand, and inducing increased production of osteoprotegerin (OPG) [21]. An inverse correlation between leptin concentration and bone mineral density (BMD) and bone turnover markers was found [22].

In healthy volunteers, a negative correlation was observed between adiponectin concentration and bone mineral density (BMD), therefore its low concentrations observed in obese persons may have positive effects on the bone formation process [23]. Other studies suggest the protective effect of adiponectin on bone tissue—this cytokine suppresses osteoclast synthesis and activates osteoblast synthesis, thus contributing to the increase of bone tissue mass [23]. The study by Thommesen et al. [24] showed that resistin activates both osteoblast proliferation and osteoclast differentiation, thus affecting bone remodelling; in addition, an inverse correlation between resistin concentrations and bone mineral density has been demonstrated [25]. This raises the question of whether liposuction procedures for purely cosmetic purposes, covering the removal of a specific volume of adipose tissue from the gluteal–femoral region, may affect the biochemical parameters of carbohydrate and lipid metabolism, and if so, what its nature is. In our study, it was observed that reduced adipokine concentrations following liposuction were accompanied by a decrease in insulin levels and HOMA-IR values. The value of the HOMA-IR under physiological conditions fluctuates around 1 and its increase may indicate the development of insulin resistance. HOMA-IR closely correlates (*r* = −0.820, *p* < 0.0001) with the insulin sensitivity index being determined based upon the standard euglycemic clamp [26,27]. It seems, therefore, that the lowering of adiponectin, leptin and resistin concentrations obtained following liposuction and the reduction of insulin resistance markers can be assessed as its positively modulating effects on fat metabolism.

There is still controversy in the literature regarding the protective effect of adipose tissue on osteoporotic changes. Few studies have reported that adipose tissue as a component of body weight and direct obesity marker has protective effects on bone tissue, reducing the risk of osteoporosis [28]; on the other hand, a negative correlation of its contents with bone mass and a higher risk of osteopenia, osteoporosis and non-vertebral fractures in subjects with higher fat content regardless of body weight [29,30]. A marker of bone metabolism, the relationship of which with adipose tissue is still sought after, is osteoprotegerin (OPG). In a few studies, significantly lower concentrations of OPG were found in the serum of obese women compared to lean women, as well as a decrease in the concentration of OPG as a result of weight reduction, which was not confirmed in the presented study [31]. In our study, no such changes were confirmed following liposuction, perhaps due to the relatively small loss in body weight and its practically normative values before the procedure. The factor significantly affecting osteoclastogenesis and bone remodelling through the effects of estrogens is TGF [32,33], and TGF-β1 is the major TGF dominant isoform in the serum and lymphoid organs [34]. TGF β has an immunosuppresive effect on T and B lymphocytes, stimulating the growth of fibroblasts [35], is a potential inhibitor of growth, which concerns, among others, bone marrow cells, hepatocytes, lymphocytes, epithelial cells of the skin or endothelial cells [36], is responsible for the growth, differentiation and migration of cells, formation and degradation of extracellular matrix components, chemotaxis and apoptosis processes playing a role in the pathogenesis of vascular inflammation, atherosclerosis [37,38].

Considering the multidirectional regulatory effects of TNF-beta, these factors have been identified as being important in assessing the metabolic consequences of liposuction. No significant changes in the concentration of this marker may be a confirmation of the lack of metabolically negatively effect of small-volume liposuction in the examined group of women. When evaluating in the presented study the effect of liposuction on other pro- and anti-inflammatory markers, an increase in Il-2 and Il-6 levels was observed, while IL-1β i Il-10 ones were unchanged in week 12 after the procedure. Pleiotropic IL-6, in 1/3 derived from adipose tissue, induces hyperglycaemia, hyperlipidaemia and insulin resistance in humans and animals [39,40]; however, is still described ambiguously in the context of osteoresorption [41,42,43]. Higher concentrations of this cytokine have been found in visceral adipose tissue, while increased levels of IL-6 precedes episodes of acute cardiovascular events and the development of type 2 diabetes [44].

The results of previous studies on the metabolic effects of liposuction are not conclusive. In the study by Klein et al. [6], in which the effect of large-volume abdominal liposuction on coronary heart disease in obese women was evaluated, it was shown that a decrease in adipose tissue mass alone does not achieve the metabolic benefits of weight loss. It should be noted that a group of 15 examined women aged 42 ± 3 years included 7 women with type 2 diabetes; therefore, this group was heterogeneous at the beginning in terms of metabolism, which certainly hindered the interpretation of the results. Subsequent published results in this area concerned the assessment of changes in selected biochemical parameters of carbohydrate and lipid metabolism as well as insulin resistance following bariatric surgery. When examining 2 women and 9 men, aged 15–58 years with a mean BMI of 45 ± 4.7 kg/m^2^, serum visfatin and TG, LDl and HDL cholesterol levels and HOMA-IR values were shown to decrease with the concomitant elevation of adiponectin [45].

Marina Yazigi Solis et al. [46] conducted a study in which the effect of physical exercise following the abdominal liposuction on serum levels and adipose tissue gene expression of selected inflammation-related adipokines in normal-weight women was analysed. In this study, an increase in TNF-α, IL-6, and IL-10 mRNA levels in subcutaneous adipose tissues was observed, while adiponectin mRNA and serum levels were decreased. The results of this study suggest that liposuction surgery may not be free of long-term metabolic effects.

## 5. Conclusions

In summary, it can be concluded that small-volume liposuction in the gluteal–femoral region in women with normative body weight does not lead to significant changes in metabolism, but only induces slight tendencies toward lowering the risk of developing insulin resistance. In the light of the heterogeneous nature of literature reports in this regard, it should be remembered that the nature of changes observed in the presented studies may be related to the selected location and, hence, to the type of removed adipose tissue. This study was not without limitations. Certainly, the relatively small group of subjects can be included among the limitations. At the same time, more distant effects than three months could be considered.

Looking further ahead, it seems reasonable to undertake comparative studies for liposuction of the gluteal–femoral and abdominal regions.

## Figures and Tables

**Table 1:** The baseline values of haematological parameters in the examined women.

Parameter	M	±Sd
Chronological age [years]	40.7	13.67
Body height [cm]	171	7.87
Lipoaspirate volume [L]	3.35	994
WBC [10^9/^L]	9.6	1.03
LYM [%]	26.38	5.52
MON [%]	5.76	1.57
NEU [%]	63.62	5.25
EOS [%]	3.34	1.95
BASO [%]	0.96	0.1
RBC [10^12^/L]	4.54	0,22
HGB [mmol/L]	13.7	0.5
HCT [mmol/L]	41.69	1.12
MCV [fl]	90.91	3.72
MCH [fmol]	29.98	1.15
MCHC [mmol/L]	33.01	0.7
PLT [10^9^/L]	334.6	23.59
ESR [mm/h]	3.9	1.2

**Table 2:** Anthropometric characteristics of the patients before and 3 months after liposuction surgery.

Parameter	Before Liposuction		After Liposuction		Wilcoxon Test *p* Value
	Median	Q25 Q75	Median	Q25 Q75	
Waist circumference [cm]	84.0	80.0 88.0	82.0	78.0 86.0	** ↧ *p* = 0.00136
Hip circumference [cm]	96.0	93.0 98.0	93.0	90.0 98.0	**↧ *p* = 0.00335
WHR	0.86	0.84 0.90	0.84	0.82 0.89	**↧ *p* = 0.00765
Body weight [kg]	68.0	63.0 73.0	64.0	60.0 70.5	**↧ *p* = 0.00145
BMI	25.9	23.1 26.0	24.5	22.0 25.0	**↧ *p* = 0.00765
PBF%	28.1	26.3 29.2	27.9	24.2 28.0	**↧ *p* = 0.00147
FAT [kg]	20.1	18.9 26.6	18.8	18.1 24.1	**↧ *p* = 0.00135
SFM [kg]	18.8	16.3 21.5	17.3	15.0 19.5	**↧ *p* = 0.00142
VFM [kg]	2.3	1.8 3.1	2.2	1.5 2.8	**↧ *p* = 0.00152

*significance level of *p* < 0.05 **: significance level of *p* < 0.01.

**Table 3:** Changes in blood biochemical parameters in response to small-volume liposuction of subcutaneous abdominal fat.

Parameter	Before Liposuction		After Liposuction		
	Median	Q25 Q75	Median	Q25 Q75	Wilcoxon Test *p* Value
Biochemical parameters					
Albumin [g/L]	28.4	26.7 29.9	28.6	28.2 33.2	*p* = 0.05821
Total protein [g/L]	36.5	30.6 37.7	38.7	34.1 39.8	*p* = 0.06217
Glucose [mmol/L]	4.6	3.4 4.2	4.7	3.5 4.3	*p* = 0.09391
Uric acid [mmol/L]	0.14	0.12 0.18	0.16	0.14 0.21	** ↥ *p* = 0.00131
Lipid profile					
TCh [mmol/L]	4.39	3.95 5.09	4.15	3.99 4.74	* ↧ *p* = 0.0277
HDL [mmol/L]	1.10	1.04 1.18	1.04	0.99 1.14	*p* = 0.13662
LDL [mmol/L]	3.31	2.97 3.43	2.99	2.93 3.89	*p* = 0.80632
TG [mmol/L]	1.55	1.44 1.62	1.44	1.37 1.62	*p* = 0.43271
ApoA [µmol/L]	81.61	69.89 83.64	79.71	64.14 89.61	*p* = 0.14182
ApoB [µmol/L]	1.65	1.58 1.71	1.61	1.54 1.71	*p* = 0.05798
ApoE [mmol/L]	1.43	1.34 1.65	1.52	1.34 1.60	*p* = 0.38255
Adipocytokines					
Adiponectin [µg/mL]	15.20	10.35 16.36	13.56	9.16 15.58	*↧ *p* = 0.03741
Leptin [µg/mL]	5.51	4.32 5.67	4.63	5.34 7.30	**↧ *p* = 0.01074
Resistin [ng/mL]	26.79	19.10 27.98	25.81	19.37 27.10	*↧ *p* = 0.03304
Visfatin [ng/mL]	4.68	3.81 4.84	4.43	3.21 4.64	*p* = 0.31084
Insulin resistance markers					
Insulin [pmol/L]	75.19	58.55 84.83	62.55	55.74 72.58	**↧ *p* = 0.00121
HOMA-IR	3.31	2.18 4.78	2.02	1.92 3.93	**↧ *p* = 0.01590
Pro- and anti-inflammatory markers					
Hs-CRP [nmol/L]	0.81	0.64 1.51	0.92	0.52 1.68	*p* = 0.22122
IL-1β [pg/mL]	7.99	7.80 8.24	7.35	6.19 7.71	*p* = 0.13256
IL-2 [pg/mL]	20.04	15.53 26.50	22.03	22.03 28.00	*↥ *p* = 0.02770
IL-10 [pg/mL]	13.42	12.98 14.17	14.08	13.00 14.99	*p* = 0.27893
IL-6 [pg/mL]	14.20	12.63 17.75	15.00	12.94 21.30	*↥ *p* = 0.03924
TNF-β [pg/mL]	214.00	198.23 273.10	212.90	202.10 263.00	*p* = 0.46361
TNF-α[pg/mL]	213.40	198.23 256.20	224.00	189.04 244.01	*p* = 0.80633
Bone turnover markers					
TGF-β1 [ng/mL]	7.20	6.43 9.01	7.67	6.41 9.62	*p* = 0.13279
OPG [pg/mL]	133.3	130.6 135.5	135.2	125.6 136.2	*p* = 0.72675

**significance level of *p* < 0.05 **: significance level of *p* < 0.01.
